# Recurrent Vertigo: Is it Takayasu's Arteritis?

**DOI:** 10.1155/2013/851352

**Published:** 2013-02-26

**Authors:** Tiwari Ashutosh, Kumar Nilesh, Varshney Ankur Nandan, Behera Dibyaranjan, Anand Arvind, Anand Ravi, N. K. Singh

**Affiliations:** Department of General Medicine, Institute of Medical Sciences, Banaras Hindu University, Uttar Pradesh, Varanasi 221005, India

## Abstract

Takayasu's arteritis (TA) is a chronic, idiopathic, inflammatory disease, that is more common in females and Asian countries. A 38-year-old female presented with recurrent vertigo. Detailed examination revealed discrepancies in peripheral pulses and raised blood pressure in bilateral lower limbs. Possibility of vasculitis involving arch of aorta or its branches was kept. Investigations were suggestive of Takayasu's arteritis, and noncontrast tomographic scanning (NCCT) of head showed B/L parietal infarcts. The disease is itself uncommon, and the presentation with vertigo only is rare. In this case vertigo may be due to Takayasu's arteritis itself or due to bilateral parietal infarcts.

## 1. Introduction

Takayasu's arteritis, formerly known as “pulseless disease,” is a chronic idiopathic inflammatory disease which affects the vessels in the body. First described in the 1800s, this rare condition is more commonly found in women in their 40s and more common in Asian countries. The aorta and its main branches are the primary vessels involved, with the most common features reflected as ischemia or aneurysm formation. The diffuse nature of this vasculitis can involve multiple organ systems to varying degrees and can present with a wide range of symptoms [1]. With Takayasu's arteritis being a rare condition and its acute phase presentation often mimicking to other conditions, diagnosis is often difficult. Diagnosis is usually guided by ACR criteria [2]. 

## 2. Case Report 

**Figure 1:**
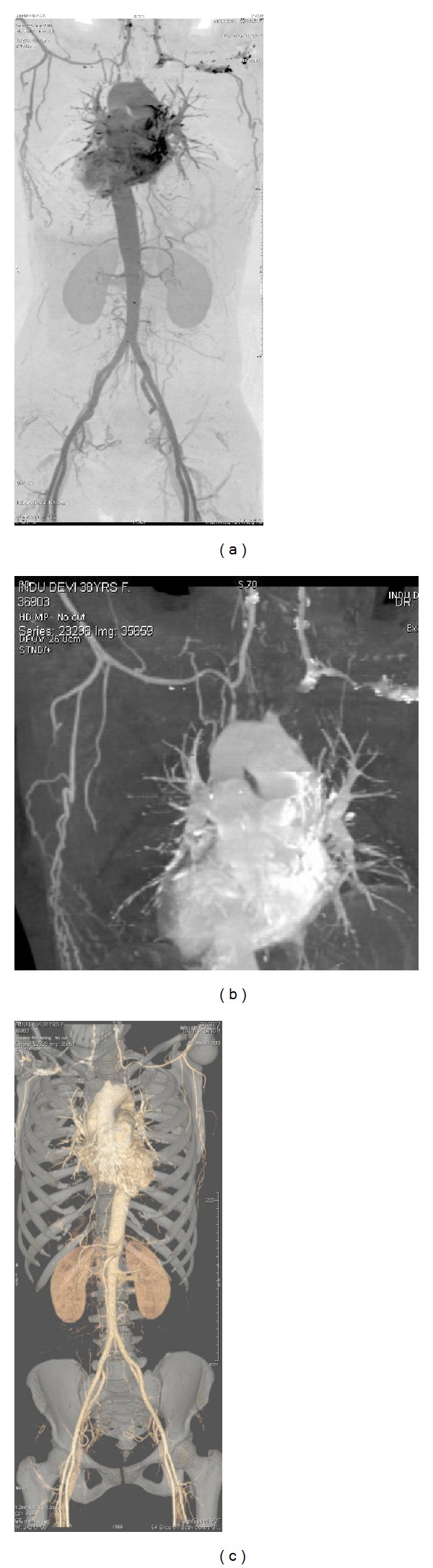
Narrowing of the origins of the great vessels with wall thickening and narrowing of great vessels for variable distance. Involvement of left subclavian artery > right subclavian artery. Involvement of right common carotid artery (CCA) > left CCA. Involvement of thoracic aorta. F/S/O Takayasu's arteritis.

A 38-year-old female presented to emergency department with the complaint of vertigo for last 3 days (only while standing and walking) and loose stools for one day, along with the complaint of similar episodes of vertigo for last 3 months which were associated with episodes of fall at times (but no loss of consciousness). On examination radial pulses were not palpable and brachial pulses were very feeble in bilateral upper limbs with no audible bruit over carotids. Detailed examination revealed palpable popliteal and dorsalis pedis arteries bilaterally in lower limbs. Her BP was recorded 194/102 mm, Hg (right lower limb), and 192/106 mm, Hg (left lower limb); examination of other systems was within normal limits except fundus examination which was suggestive of hypertensive changes. Possibility of vasculitis involving aorta and its branches was kept and investigations were sent. Routine investigations were within normal limits. Lipid profile, 2D echo, was normal. Anti-nuclear antibody, anti-cardiolipin antibody, anti-phospholipid antibody, anti-beta-2-glycoprotein antibody, and lupus anti-coagulant were negative. ESR and CRP were raised (52 mm/hour and 27.6 mg/L resp.). NCCT head showed ischemic infarcts in bilateral parietal lobes. CT angiography showed narrowing of right brachiocephalic trunk, right subclavian artery, part of right common carotid, left common carotid, and subclavian artery (Figures 1(a), 1(b), and 1(c)).

## 3. Discussion 

Takayasu's arteritis is a chronic, inflammatory disease of unknown etiology that primarily affects large blood vessels such as aorta and its branches [3]. Vertigo is a common complaint but it is less reported with Takayasu's arteritis and rarely reported as the sole presentation of this disease. We report such a case and emphasize the need for thorough evaluation of patients with vertigo to establish the underlying etiology. In 1908 Takayasu's, a Japanese ophthalmologist, first described the clinical entity in a young female with retinal changes. In 1928 Sharma et al. described it as pulseless disease [4]. The first autopsy on a patient with TA was carried out in 1940 by Ohta [5]. Its estimated incidence is 2.6/100,000,0/year [3]. The disease has worldwide distribution but is more common in Japan, India, and China [6]. It affects predominantly females, with a male to female ratio of 1 : 9 but the difference is much less in India, where the male to female ratio is 1 : 1.5–32. Pathogenesis of the disease remains unclear but there is an indirect evidence of an autoimmune process. Takayasu's arteritis has a variable presentation. About 15–50% of the patients have nonspecific symptoms like fever, malaise, myalgia, weight loss, and arthralgia. One-third of the patients are anemic and 10–30% have various cardiac symptoms. It is the most common cause of renovascular hypertension in India. About half of the patients have neurologic symptoms, with the most common being visual symptoms. Strokes occurring in 10% of the patients [6]. The commonest sites of involvement are the basal ganglia and watershed zones [7]. Diagnosis is based on clinical features and angiographic findings. 

## 4. Conclusion 

With this case report we want to give a message that even a major and rare disease can present with a minor or nonspecific complaint. And thorough evaluation for the possible complications of a disease may reveal the occult damages. As in the above case, a patient of Takayasu's arteritis presented with the complaint of recurrent vertigo, and parietal infarcts were detected in NCCT. Vertigo would have been caused due to Takayasu's arteritis related narrowing of the vessels supplying to brain or it may be due to parietal infarcts with which it is rarely reported.
